# Downregulation of miR-322 promotes apoptosis of GC-2 cell by targeting Ddx3x

**DOI:** 10.1186/s12958-019-0506-7

**Published:** 2019-08-05

**Authors:** Qi Che, Wei Wang, Peng Duan, Fang Fang, Chunyan Liu, Ting Zhou, Honggang Li, Chengling Xiong, Kai Zhao

**Affiliations:** 1 Family Planning Research Institute/Center of Reproductive Medicine, Tongji Medical College, Huazhong University of Science and Technology, Wuhan, 430030 China; 2 Wuhan Tongji Reproductive Medicine Hospital, Wuhan, 430014 China

**Keywords:** miR-322, Ddx3x, GC-2 cell, Apoptosis

## Abstract

**Background:**

Aberrant DNA damage of germ cells, which impairs spermatogenesis and lowers fertility, is an important factor contributing to male infertility. MicroRNAs (miRNAs) play a significant role in the expression and regulation of multiple genes during spermatogenesis. Our previous study found much lower miR-424 (murine homologue miR-322) levels in the seminal plasma of infertile patients with high DFI(DNA Fragmentation Index)than in the fertile group. However, the mechanism by which miR-322 regulates germ cells during spermatogenesis remains unknown.

**Methods:**

In this study, we successfully established a GC-2 cell model of miR-322 downregulation resulting in impaired spermatogenesis. And the cell viability were measured using Cell Counting Kit-8 (CCK-8; Dojindo, Japan) and MTT (Sigma Aldrich, USA). Immunofluorescence assay was used to detect cell damage and the expression of apoptosis-related proteins were measured using real-time quantitative PCR and Western blot analysis. Target genes were predicted and verified by online database retrieval and Dual-luciferase reporter gene assay.

**Results:**

We observed evident decreases in the cell viability of GC-2 cells along with remarkable increases in apoptosis after miR-322 inhibition. While the expression of apoptosis-related genes, including Bax and caspases 3, 9, and 8 greatly increased in GC-2 cells after miR-322 downregulation, that of the anti-apoptotic Bcl-2 gene decreased. Ddx3x was found to be the direct target of miR-322. MiR-424 was then detected in the seminal plasma of infertile patients with high DFI(DNA Fragmentation Index); this miRNA was down-regulated but Ddx3x was upregulated in the infertile group.

**Conclusion:**

MiR-322 plays a key role in promoting GC-2 cell apoptosis by directly regulating Ddx3x expression. MiR-424 downregulation in infertile men may induce spermatogenic cell apoptosis and sperm DNA damage by directly acting on the target gene locus Ddx3x, resulting in male infertility.

**Electronic supplementary material:**

The online version of this article (10.1186/s12958-019-0506-7) contains supplementary material, which is available to authorized users.

## Introduction

The integrity of sperm DNA is vital to the outcome of pregnancy [1]. The ability of sperm to penetrate the egg is reduced when sperm DNA is damaged, thereby affecting the development of the embryo and implantation, reducing the pregnancy rate, and increasing the spontaneous abortion rate [2, 3]. Sperm DNA damage frequently occurs in the male gamete of infertiles [4] due to various factors, including anomalous sperm chromatin assembly [5], oxidative stress [6], and sperm cell apoptosis [5].

MicroRNAs (miRNAs) are a small group of non-coding RNAs. Mature miRNAs produced and matured by specific endonucleases from the stem-loop structures of approximately 70 nucleotides [7] are small single-strand RNAs of 19–23 nucleotides. The spatial and temporal expressions of miRNAs are involved in the regulation of many biological processes, such as cell proliferation, cell differentiation, and apoptosis [8]. Several studies have suggested that miRNAs play a crucial role in regulating spermatogenesis. For instance, mouse knockouts of Dicer1 present few tubules containing elongated spermatids [9, 10]. Dicer1 gene can produce Dicer enzyme, which processes microRNA precursors into their mature form, enabling them to regulate gene expression. MiR-146 is reported to modulate the effects of retinoic acid on spermatogonial differentiation in mice [11]. Ccnt2, one of the targets of miR-15a, plays a regulatory role in early spermatogenesis. MiR-122 may influence spermatozoa-like cells by suppressing TNP2 expression and inhibiting the expression of proteins associated with sperm development [12], while miR-34c enhances murine male germ cell apoptosis through targeting ATF1 [13].

Our previous study found low miR-424 (murine homologue miR-322) expression, which could cause sperm DNA damage [14], in the seminal plasma of infertile patients. Overexpression of miR-322 enhances the expression of Osterix and other osteogenic genes, whereas the target gene of miR-322, Tob2, functions as a negative regulator of osteogenesis by mediating Osterix mRNA [15] degradation. Evidence that the miR-322 mimic and knockdown of TRAF3 as the target gene of miR-322 block apoptosis has been obtained, and the miR-322 inhibitor has been observed to mimic the effect of high glucose, leading to apoptosis in neural stem cells [16]. In the human neuroblastoma cell line, miR-322 may attenuate apoptosis by recovering the change in expression of c-Myc17. While the regulatory function of miRNA-322 in many other cells has been demonstrated, few studies focusing on its role in spermatogenesis have been published. The regulation of target genes is known as a key point in modulating various biological processes, among which regulating apoptosis miRNAs are functionally important [17]. Thus, to investigate the contribution of miR-322 to spermatogenesis regulation, we developed a GC-2 cell model of miR-322 downregulation to explore the potential targets of miR-322 and directly verify our hypothesis that miR-322 regulates the apoptosis of germ cells.

## Materials and methods

### Cell culture

GC-2spd(ts)was established by stable cotransfection of freshly isolated spermatocytes with the SV40 large T antigen gene (pSV3neo, see ATCC 37150) and the cell type is spermatocyte. Adherent GC-2 (ATCC, USA) cells were cultured in high-glucose Dulbecco’s Modified Eagle’s Medium (Gibco, USA) containing 10% fetal bovine serum (Gibco, USA) and 1% antibiotics (100 U/ml penicillin and 100 mg/ml streptomycin, Life Technology Inc., USA). Cells were incubated at 37 °C in a humidified incubator with 5% carbon dioxide and then passaged via trypsinization (trypsin-EDTA; Gibco, USA) thrice a week.

### Cell transfection and cell model establishment

MiR-322 inhibitors and miRNA negative controls (NCs) were purchased from RiboBio (Guangzhou, China). GC-2 cells were seeded in a 96-well plate at a cell density of 1 × 10^3^ cells per well or a six-well plate at 1 × 10^5^ cells per well for 24 h before transfection. Prior to miRNA transfection, the cell culture medium was replaced with serum-reduced medium (Opti-MEM; Invitrogen, USA). Lipofectamine 2000 (Invitrogen, USA) was used to transfect GC-2 cells. Cells were incubated with miR-322 inhibitors or an NC/Lipofectamine mixture for 6 h. Then, the Opti-MEM I medium was switched to the cell culture medium of GC-2 cells. All transfection procedures were performed according to the protocols supplied by manufacturers. To determine optimal transfection conditions, Gmr-mirtm FAM labeled microRNA inhibitors control was used to detect transfection efficiency and intracellular distribution of microRNA inhibitor. The cell group with the lowest expression of miR-322 was selected as the GC-2 cell model of miR-322 inhibition.

### Cell viability assay

MTT (Sigma Aldrich, USA) proliferation assay and Cell Counting Kit-8 (CCK-8; Dojindo, Japan) were used to detect cell viability. For the MTT assay, transfected GC-2 cells were washed twice with PBS (HyClone, USA). A total of 25 μl of MTT (50 mg/ml) was added to each well, and the cells were incubated at 37 °C for 4 h. Then, the culture medium of each well was replaced with 150 μl of dimethyl sulfoxide (Sigma) and shaken for 15 min. The absorbance was measured at 490 nm by a microplate reader (Thermo). For CCK-8, after washing the model GC-2 cells twice with PBS, 10 μl of CCK-8 solution was added to each well. The plates were then incubated at 37 °C for 4 h, and the absorbance at 450 nm was measured by a microplate reader.

### Apoptosis assay

Apoptosis evaluation of GC-2 cell was performed by an FITC Annexin V Apoptosis Detection Kit with 7-AAD (BioLegend, USA) according to the manufacturer’s instructions. After harvesting the model GC-2 cells, they were washed with ice-cold PBS twice. Annexin V binding buffer was used to resuspend cells to the concentration of 0.25~1 × 10^7^ cells/ml. In addition, 5 μl of FITC-Annexin V and 5 of μl 7-AAD were successively added to test tubes with 100 μl of the cell resuspension. The cells were then incubated for 15 min at room temperature in the dark. Stained cells were added with 400 μl of Annexin V binding buffer in each tube, and flow cytometry (BD, USA) was applied to analyze apoptosis. Annexin V+/7-AAD- cells were considered early apoptotic cells, while Annexin V−/7-AAD+ cells were determined as late apoptotic cells.

### RNA extraction and quantitative real-time PCR

Total RNA was extracted from the GC-2 cells using TRIzol reagent (Invitrogen, USA), and the total RNA concentration was determined by measuring the absorbance at 260 nm using a NanoDrop 2000 (Thermo Fisher Scientific, USA). cDNA synthesis of coding genes (including Bcl-2, Bax, caspases 3, 9, and 8, Ddx3x, Wee1, and Rad23b) and miRNAs (including miR-322 and miR-29c) were respectively performed using the PrimeScript RT–PCR kit (TaKaRa, Japan) and the miRNA First Strand cDNA Synthesis Kit (Sangon Biotech, China) following the standardized protocol of the manufacturers. Gene and miRNA expressions were detected with an ABI Step One System (Applied Biosystems, USA) as previously described [14]. The specific steps of real-time PCR are as follows: 0.5ul upstream primers,0.5 ul downstream primers,1.5 ul template cDNA,7.5 ul RNase Free Water, and 10 ul SyberGree dyes(TaKaRa, Japan) were added to the eight tubes, and then amplified in the real-time PCR instrument,40 cycles were performed at 95 °C min,60 °C 30s and 72 °C 30s,the CT value of internal reference was in the range of 17–22, and the CT value of target gene was in the range of 25–30, after the end of amplification, the specificity of the reaction was determined by the melting curve drawn by real-time PCR instrument, and the results were analyzed and compared with the experimental results by 2-△△ct. β-Actin and U6 were used to normalize expression levels, and all primers were synthetized by Sangon Biotech, and Additional file 2: Table S1 lists the primer sequences.

### γ-H2AX staining and immunofluorescence test

GC-2 cells were seed in 6 well dishes and make cell slides. MiR-322 inhibitor and the miRNA inhibitor NC were transfected mixed with lipo2000 respectively. 24 h after transfection, cells were fixed with 100% cold methanol at − 20 °C for 15 min, and permeabilized with 0.2% Triton X-100 for 10 min. The cells were then blocked with 3% BSA in PBS for 1 h at room temperature, and incubated overnight at 4 °C with γ-H2AX monoclonal antibody (1:500, Abcam, ab26350). After three washes, cells were incubated with the secondary antibody conjugated with Alexa Fluor 488 (Molecular Probes) for 2 h at room temperature. Then, DIPI was used for staining. Images were acquired using OlymPus BX53 Fluorescence microscope.

### Western blot

The supernatants of the cells were collected after lysing and centrifugation at 12,000 rpm for 30 min at 4 °C. The concentrations of each protein were determined using the BCA Protein Assay Kit (Thermo-Fisher) according to the manufacturer’s protocol. Protein samples (30 μg) were separated by 10% SDS–PAGE and then transferred to an equilibrated PVDF membrane (Millipore, USA). Primary antibodies (Bcl-2, Bax, caspases 3, 9, and 8, Ddx3x, Cleaved-caspases3,9 and GAPDH) were mixed with the PVDF membrane at a concentration of 1:1000 for overnight incubation on a shaker at 4 °C. Rabbit anti-mouse antibodies were used as primary antibodies and purchased from Sigma-Aldrich (USA). Recycle the diluted primary antibody, wash it with TBST three times, 5 min each;Add the diluted secondary antibody (5% skim milk diluted by 1:50,000), incubate it at room temperature for 30 min, and wash it in a shaking bed at room temperature with TBST for 4 times, 5 min each time. Chemiluminescence detection: drop the freshly prepared ECL mixed solution to the protein side of the membrane, and adjust the exposure conditions in the darkroom according to different light intensity. Analysis of development and fixation results: the film was scanned and archived, and AlphaEaseFC software processing system analyzed the optical density value of the target band.

### Target gene prediction

The possible target genes of miR-322 were predicted by searching through several online databases, including TargetScan (http://www.targetscan.org), starBase (http://starbase.sysu.edu.cn/targetSite.php), TarBase (TarBase: http://www.microrna.gr/tarbase), mircoRNA.org (http://www.microrna.org), and miRDB (http://www.mirdb.org/). One gene among the three best candidate genes was selected as the possible target gene by quantitative RT–PCR analysis.

### Dual-luciferase reporter gene assay

The 3’UTR sequence of Ddx3x mRNA containing miR-322 binding sites and the corresponding mutated sequence were respectively inserted into the XbaI and XhoI sites of the pmirGLO vector (Promega, USA) (hereinafter named pmirGLO-Ddx3x-WT and pmirGLO-Ddx3x-MT). GC-2 cells seeded in 24-well plates were co-transfected with miR-322 mimics and wild-type/mutant reporter plasmid vectors. After 24 h of transfection, cells were harvested and luciferase assays performed by using the Dual Luciferase Reporter System (Promega) following the standardized protocol of the manufacturer. The firefly luciferase activity of each measured sample was normalized to the *Renilla* luciferase activity.

### Analysis of miR-424 (murine homologue miR-322) expression and its possible target gene Ddx3x inseminal plasma of male infertility patients with high DFI

RNA and protein were extracted from the seminal plasma of 30 male sterile patients with high DFI and 30 normal males as control. Changes in miR-424 expression were detected by real-time PCR. Two patients and two normal males were selected from the experimental and control groups, and Western blot was used to detect changes in the protein expression of the possible target gene Ddx3x. Results were compared between groups.

### Statistical analysis

All experiments were independently performed at least thrice in this study, and all data are presented as the mean ± standard error of the mean (SEM). All analyses were performed using SPSS 16.0 for Windows (SPSS Inc., USA). Differences were considered significant at *P* < 0.05.

## Results

### Establishment of the GC-2 cell model of miR-322 downregulation

**Fig. 1:**
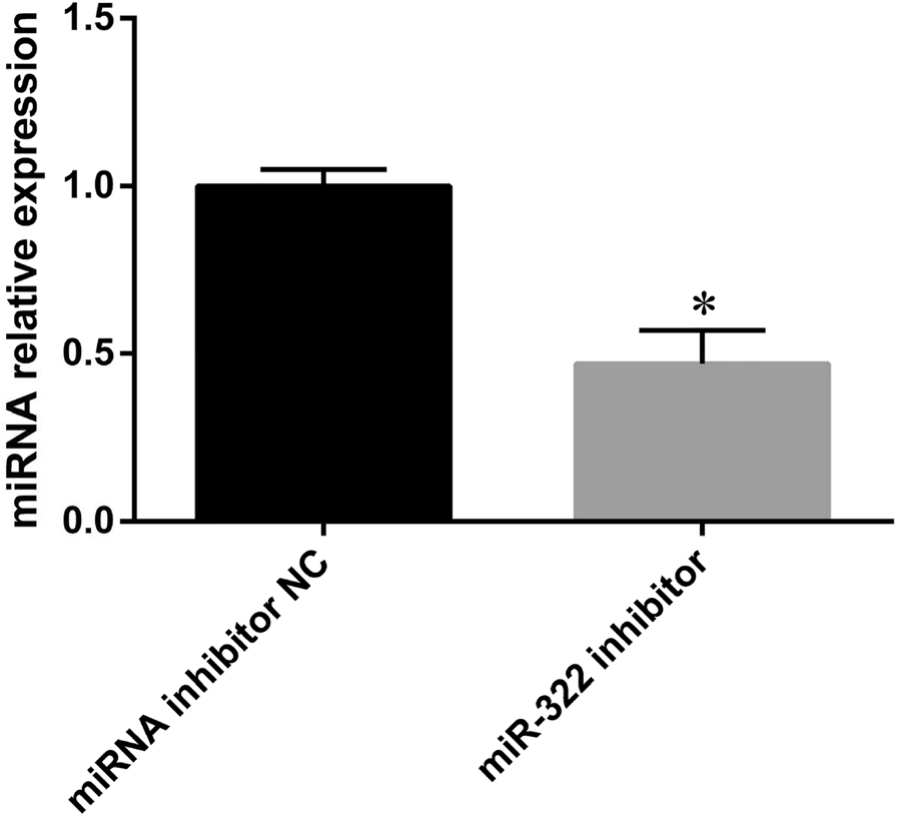
GC-2 cell model of miR-322 downregulation. The relative expression of miR-322 was tested by quantitative RT-PCR using U6 as the internal control. MiR-322 expression in GC-2 cells treated with miR-322 inhibitors was significantly decreased (1 vs 0.48, *P* < 0.05) (Fig. 1). Each bar represents the mean ± SEM of at least three independent experiments for each group (^*^P < 0.05)

In our study, miR-322 inhibitors were transfected to suppress miR-322 expression in GC-2 cells, while miRNA inhibitor NCs were transfected as the control group. MiR-322 expression in GC-2 cells treated with miR-322 inhibitors was significantly decreased (1 vs 0.48, *P* < 0.05) (Fig. 1). In subsequent analyses, GC-2 cells with miR-322 downregulation were considered the experimental group.

### MiR-322 downregulation decreased CG-2 cell viability and induced cell apoptosis

**Fig. 2:**
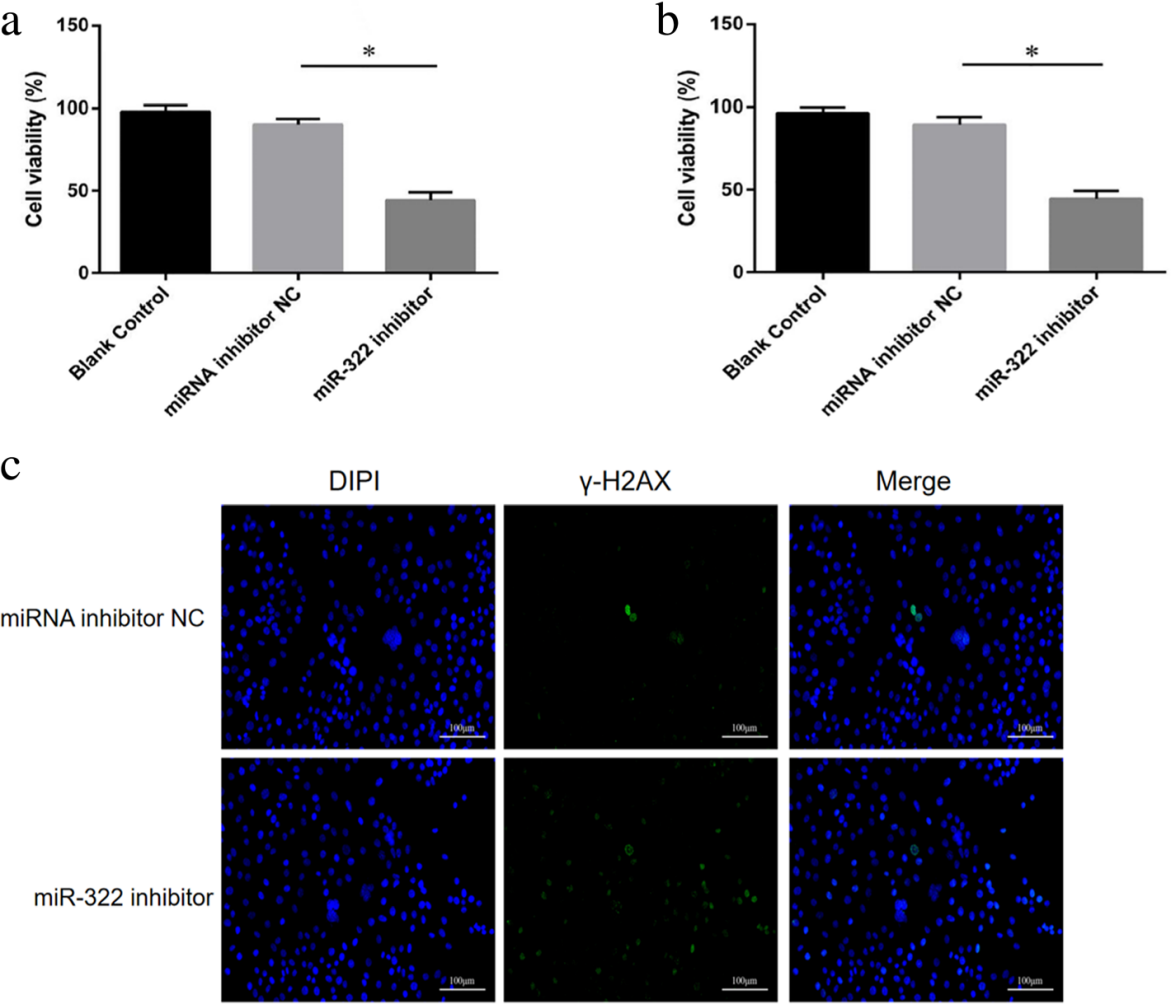
Effects of miR-322 inhibition on GC-2 cell viability. **a** MTT assay was performed to determine the viability of cells transfected with miRNA inhibitor NCs and miR-322 inhibitors. Cells without transfection were considered blank controls. **b** Results of CCK-8 assay to detect the cell viability of the miRNA inhibitor NC, miR-322 inhibitor, and blank control groups. And figs. **a** and **b** illustrate that the cell viability of the experimental group was significantly decreased (93.18% vs 46.13%; 90.85% vs 45.1%, *P* < 0.05) compared with that of the control group. **c** Cell damage was detected by immunofluorescence assay. Mir-322 inhibitor was transfected into the experimental group and the miRNA inhibitor NC was transfected into the control group.The data represent as mean ± SEM of three separate experiments (^*^P < 0.05)

**Fig. 3:**
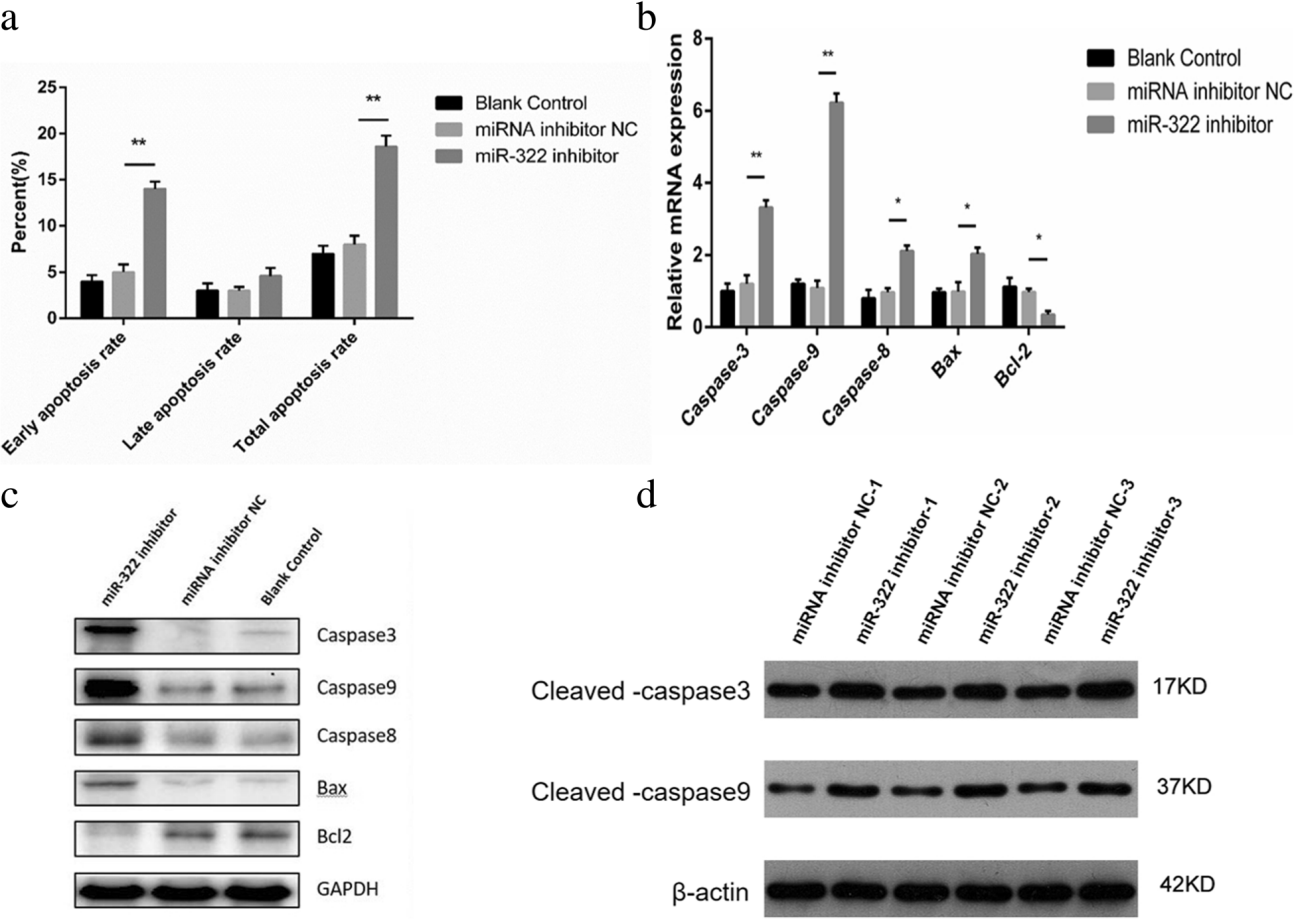
MiR-322 downregulation promoted GC-2 cell apoptosis. **a** Cell apoptosis was analyzed by flow cytometry. Early apoptosis and total apoptosis rates in the experimental group were markedly higher (5.12% vs 13.92%; 6.5% vs 17.5%) than those of the control group (*P* < 0.01) (**b**) Relative expressions of caspases 3, 9, and 8, Bax, and Bcl-2 at the mRNA level were determined by quantitative RT-PCR using β-actin as the internal control. **c**, **d** Relative expressions of caspases 3, 9, and 8, Cleaved caspases3 and 9,Bax, and Bcl-2 at the protein level were determined by Western blot using GAPDH as the internal control. Compared with the control group, the mRNA expression levels of caspases 3, 9, 8 and Bax in the experimental group were 1.27% vs 3.37,1.11% vs 6.37,0.95% vs 2.22 and 1.04% vs 2.15%, respectively. by contrast, Bcl-2 expression significantly decreased (1.02% vs 0.33%,*P* < 0.05) compared with that in the control group.Each bar presents the mean ± SEM of three independent experiments for each group (^*^P < 0.05, ^**^ *P* < 0.01)

Figure 2a and b were detected by MTT and cck-8 methods, respectively. And Fig. 2a and b illustrate that the cell viability of the experimental group was significantly decreased (93.18% vs 46.13%, 90.85% vs 45.1%,P < 0.05) compared with that of the control group. According to the results of Fig. 2c, the number of fluorescence focus of GC-2 cells in the miR-322 inhibitor group was higher than that in the miRNA inhibitor NC group, indicating increased cell damage after knockdown of miR-322. Furthermore, early apoptosis and total apoptosis rates in the experimental group were markedly higher (5.12% vs 13.92%, 6.5% vs 17.5%) than those of the control group(*P* < 0.01) (Fig. 3a). No evident difference in late apoptosis rate (2.25% vs 4.585% ,*P* > 0.05) was found between both groups. These data indicate that miR-322 downregulation promote early apoptosis of GC-2 cells.

### MiR-322 downregulation affected the expression of cell apoptosis factors in GC-2 cells

To confirm the effects of miR-322 downregulation on GC-2 cell apoptosis, the expression of apoptosis factors (Bcl-2, Bax, and caspases 3, 9, and 8) was examined. Figure 3b, c and d show that the expressions of Bax, caspases 3, 9, and 8 and Cleaved caspases3 and 9 significantly increased at the mRNA and protein levels in the experimental group (*P* < 0.05); Compared with the control group, the mRNA expression levels of caspases 3, 9, 8 and Bax in the experimental group were 1.27% vs 3.37,1.11% vs 6.37,0.95% vs 2.22 and 1.04% vs 2.15%, respectively. by contrast, Bcl-2 expression significantly decreased (1.02% vs 0.33% ,*P* < 0.05) compared with that in the control group.

### Ddx3x is the direct target of miR-322

**Fig. 4:**
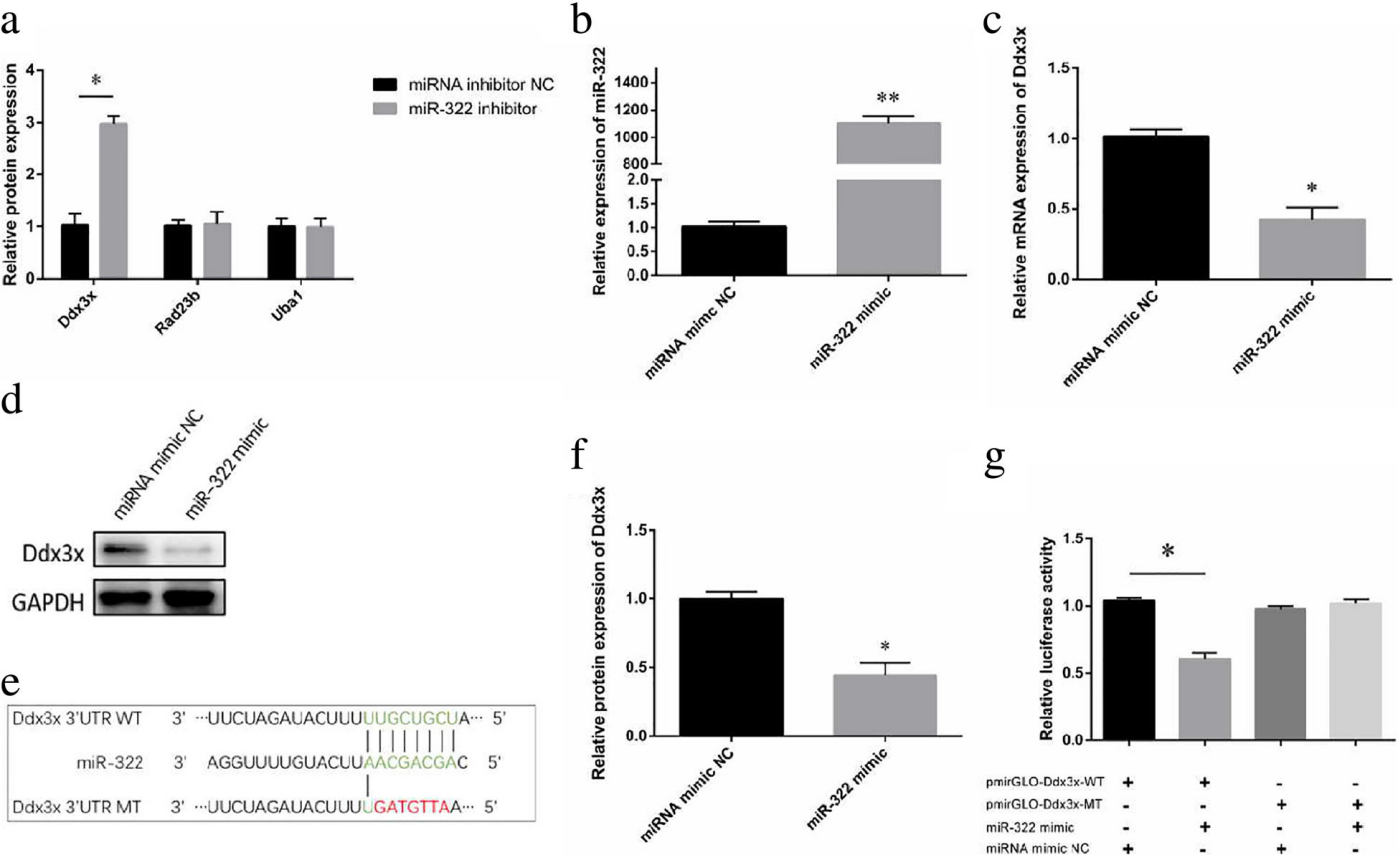
Ddx3x is the direct target of miR-322. **a** Quantitative RT–PCR was performed to evaluate the expression of three possible candidate genes using β-actin as the internal control.The relative expression of Ddx3x significantly decreased (1 vs 3, *P* < 0.05) compared with that of the two other candidate genes, as shown in 4**a**. **b** MiR-322 expression was evaluated by real-time PCR after transfection with miR-322 mimics or miRNA mimic NCs using U6 as the internal control. **c** The relative mRNA expression of Ddx3x after miR-322 inhibition was detected by quantitative RT-PCR using β-actin as the internal control. **d**, **f** The relative protein expression of Ddx3x after miR-322 inhibition was determined by Western blot using GAPDH as the internal control.Further analysis revealed that miR-322 expression significantly increased (1 vs 1100; *P* < 0.05) whereas Ddx3x expression significantly decreased (1 vs 0.43; 1 vs 0.4, *P* < 0.05) in GC-2 cells transfected with miR-322 mimics (**b–d** and **f**). **e** Sequence alignment of putative amino acids for miR-322 and the 3’UTR of Ddx3x (Ddx3x 3’UTR-WT). The mutant, namely, Ddx3x 3’UTR-MT, is underlined. **g** MiR-322 directly targeted Ddx3x. Luciferase reporters containing either Ddx3x 3’UTR-WT or Ddx3x 3’UTR-MT were co-transfected with miR-322 mimics or miRNA mimic NCs into GC-2 cells. Reporter activity significantly decreased (1.05 vs 0.58) after miR-322 overexpression compared with the control. All data represent the mean ± SEM of at least three independent experiments (*P < 0.05, ** *P* < 0.01)

Three genes (Ddx3x, Uba1, and Rad23b) that appeared to be the most likely target of miR-322 among five databases (i.e., TargetScan, starBase, TarBase, mircoRNA.org, and miRDB) were identified as candidate genes. The relative expression of Ddx3x significantly decreased (1 vs 3, *P* < 0.05) compared with that of the two other candidate genes, as shown in Fig. 4a. We thus suspect that Ddx3x is the target gene of miR-322. Further analysis revealed that miR-322 expression significantly increased (1 vs 1100, *P* < 0.05) whereas Ddx3x expression significantly decreased (1 vs 0.43, 1 vs 0.4, *P* < 0.05) in GC-2 cells transfected with miR-322 mimics (Fig. 4b–d and f).

To confirm whether the 3’UTR region of Ddx3x could be directly bonded by miR-322, wild type and mutant luciferase reporter plasmids containing the binding region of the 3’UTR of Ddx3x mRNA were established (Fig. 4e). Compared with that in the control group, luciferase activity significantly decreased in the group of co-transfected with miR-322 mimics and pmirGLO-Ddx3x-WT reporter plasmids in GC-2 cells (1.05 vs 0.58, *P* < 0.05) (Fig. 4g). These results suggest that miR-322 could directly target Ddx3x.

### Upregulation of miR-424 and its possible target gene Ddx3x decreased in the seminal plasma of male infertility patients with high DFI

**Fig. 5:**
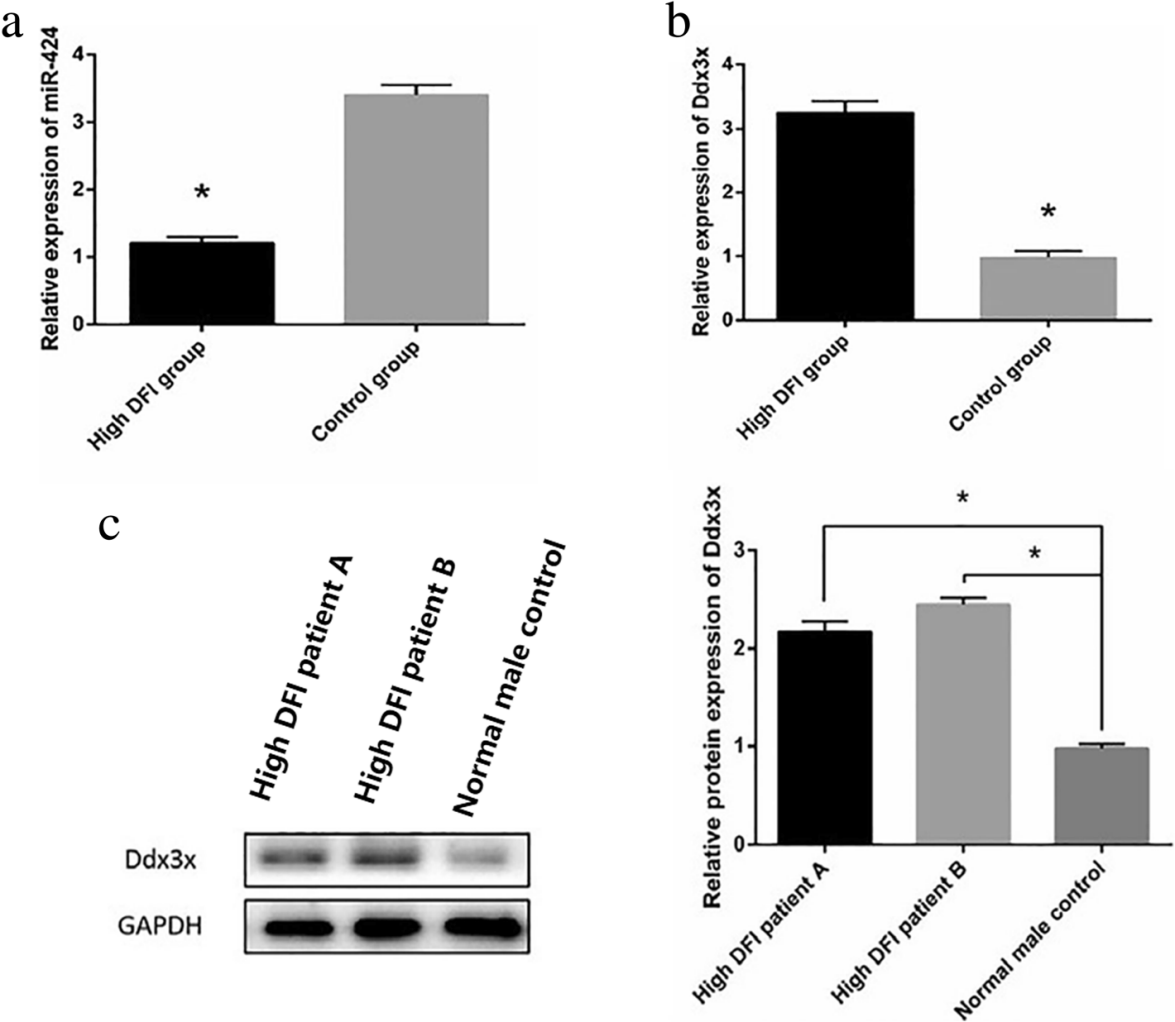
Changes in mir-424 and Ddx3x in the sperm plasma of males with high DFI infertility. **a** The relative expression of miR-424 was tested by quantitative RT-PCR using U6 as the internal control.miR-424 expression in seminal plasma of the group with high DFI male infertility was significantly reduced (1.14 vs 3.26). **b** The relative expression of Ddx3x was tested by quantitative RT-PCR using U6 as the internal control. **c** The relative expression of Ddx3x at the protein level was determined by Western blot using GAPDH as the internal control. **b** (3.14 vs 0.95) and **c** (2.1 or 2.4 vs 1) also reveal that, as the target gene locus of mir-322 in mice and the most likely target gene locus of mir-424 in the seminal plasma of male infertility patients with high DFI, Ddx3x showed a significant increase in mRNA (3.14 vs 0.95) and protein (2.1 or 2.4 vs 1) levels . All data represent the mean ± SEM of at least three independent experiments (*P < 0.05, ** *P* < 0.01)

Compared with that in the normal group, miR-424 expression in seminal plasma of the group with high DFI male infertility was significantly reduced (Fig. 5a;1.14 vs 3.26, *P < 0.05). This result is consistent with our previous finding that miR-424 is differentially and poorly expressed in the seminal plasma of male infertility patients with high DFI. Figure 5b (3.14 vs 0.95) and c (2.1 or 2.4 vs 1) also reveal that, as the target gene locus of mir-322 in mice and the most likely target gene locus of mir-424 in the seminal plasma of male infertility patients with high DFI, Ddx3x showed a significant increase in mRNA (3.14 vs 0.95) and protein (2.1 or 2.4 vs 1) levels (*P < 0.05).

## Discussion

Based on previous studies, we hypothesized that miR-322 inhibition promotes apoptosis in GC-2 cells. In the present research, we observed a significant decrease in cell viability (*P* < 0.05) and increase in apoptosis in the GC-2 cell model of miR-322 downregulation. The trends of expression changes in apoptosis factors (caspases 3, 9, and 8, Bax, and Bcl-2) were consistent with that of apoptosis promotion. Furthermore, Ddx3x was verified to be a direct target of miR-322 regulating GC-2 cell apoptosis. Taken together, these data preliminarily demonstrate the effects of miR-322 inhibition on GC-2 cell apoptosis in the male reproductive system.

While many miRNAs are identified as negative regulators in many biological cell processes, other miRNAs can play positive roles in cell growth, differentiation, and proliferation. For example, miR-322 suppresses the expression of specific target genes that originally have a negative regulation function. Previous studies have demonstrated the vital role of miR-322 in cell growth and apoptosis [15, 18]. However, research focusing on the regulation function in the reproductive system is scarce.

Our previous study indicated that miR-322 is downregulated in mouse seminal plasma and may lead to sperm DNA damage [14]. In the present research, we developed a model of miR-322 downregulation using GC-2 cells by transfecting miR-322 inhibitors to simulate this dysregulation. To exclude the interference of miR-29c downregulation, which may lead to the same damage, we also established a GC-2 cell model of miR-29c downregulation in the same manner by referencing our previous results. Cell viability was examined using the same method, and the results excluded the possibility of the interfering effect of miR-29c (Additional file 1: Figure S1, 1 vs 0.5; Sb, 96.9 and 95.37% vs 90.73%; Sc, 96.9 and 90.73% vs 87.64%).

Consistent with our previous hypothesis, significant declines in cell viability and increases of apoptosis induced by miR-322 downregulation were observed. Subsequent results revealed that relative caspase 3, 9, and 8, and Bax expressions were all upregulated whereas that of Bcl-2 was downregulated at the mRNA and protein levels. The expressions of these apoptosis factors at both levels indicates that GC-2 cells with miR-322 downregulation result in apoptosis while suppressing cell proliferation.

Among the total identified caspases in our study, changes in caspase 3 and 9 stood out as the most significant. Past research reveals that, as the direct target of miR-98, caspase-3 induces apoptosis via the loss of mitochondrial membrane potential and caspase activation [19]. Moreover, as the target of miR-133, caspase-9 is suppressed in the apoptosis of myocardiocytes [20]. The intrinsic (mitochondrial) and extrinsic (death receptor) pathways are the two main pathways of cell apoptosis [21]. Caspases 3 and 9 are closely connected vital factors in their regulatory networks. We suggest that caspase-9 is required to activate all downstream caspases, including caspases 2, 3, 6, 7, 8, and 10. Caspase-3 is also required for the feedback amplification loop involving caspase-9 [22]. Our findings agree with these changes in relative factors inducing apoptosis activation.

In this study, we initially predicted and then confirmed that Ddx3x is the direct regulation target gene of miR-322 in the activation of GC-2 cell apoptosis.

As a member of the highly conserved family of DEAD-box RNA helicase, Ddx3x features homologous genes in various eukaryotes and is highly expressed in the testis, similar to Ddx3y. Ddx3x participates in regulating many physiological functions, including transcription, RNA cleaving and modification, and initiation of translation and apoptosis [23]. Therefore, Ddx3x is essential for the management and regulation of normal spermatogenesis. Existing studies on Ddx3x mainly focus on its modulatory role in the infection of HIV and HCV and the generative mechanism of complications after hepatitis B and C infection [24]. In addition, Ddx3x is suggested to affect cancer cell motility and proliferation [25], including lung [26], colon, and breast cancer [26]. Moreover, Ddx3x plays an important role in the regulation of cell mitosis [27].

Previous study revealed that high DDX3 levels in cells with p53 permit extensive activation of the extrinsic apoptosis pathway after DNA damage. As knocking down DDX3 reduces p53 levels and DDX3 overexpression increases p53 levels following DNA damage, DDX3 apparently participates in the regulation of p53 accumulation following DNA damage of cells containing functional p53 through caspase activation [28]. During mouse early embryonic development, Ddx3x regulates cell survival and apoptosis by accumulatingp53 in blastocysts [29]. Interestingly, Ddx3x knockout in mouse results in embryonic lethality, and the observation of significantly increased γH2AX and p53 indicates DNA damage, which emphasizes the necessity of Ddx3x for both embryo and extraembryonic development [30]. Considering the studies presented here, we suspect that Ddx3x regulates a range of functions in cell survival and apoptosis. However, this hypothesis requires further investigation.

## Conclusions

Our results initially demonstrated that miR-322 lowers cell viability when inhibited in GC-2 cells. Downregulated miR-322 may promote GC-2 cell apoptosis with attenuation of Bcl-2 and activation of caspases 3, 9, and 8 and Bax. Moreover, Ddx3x appeared to be the directly regulated gene target of miR-322 to induce apoptosis. Although our findings identified miR-322 as the modulator of GC-2 cell apoptosis, the detailed molecular mechanism requires further examination.

## Additional files

Additional file 1: **Figure S1.** Effects of miR-29c inhibitor transfection on GC-2 cell apoptosis. (a) The relative expression of miR-29c was measured by quantitative RT-PCR using U6 as the internal control. (b) MTT assay was performed to evaluate cell viability after miR-29c inhibition. Cells without transfection were considered blank controls. (c) CCK-8 assay was performed to evaluate cell viability after miR-29c inhibition. Cells without transfection were considered blank controls. All the results excluded the possibility of the interfering effect of miR-29c (Sa, 1 vs 0.5; Sb, 96.9 and 95.37% vs 90.73%; Sc, 96.9 and 90.73% vs 87.64%). All data represent the mean ± SEM of at least three independent experiments (^*^P < 0.05). (PNG 453 kb)

Additional file 2: **Table S1.** Sequences of primers for quantitative RT-PCR. (DOCX 43 kb)

## Data Availability

The dataset supporting the conclusions of this article is included within the article.
