# Supplementary figures and images for: Downregulation of miR-322 promotes apoptosis of GC-2 cell by targeting Ddx3x
Source: Reprod Biol Endocrinol. 2019 Aug 5;17:63. doi: 10.1186/s12958-019-0506-7 (PMC6683552; doi:10.1186/s12958-019-0506-7)

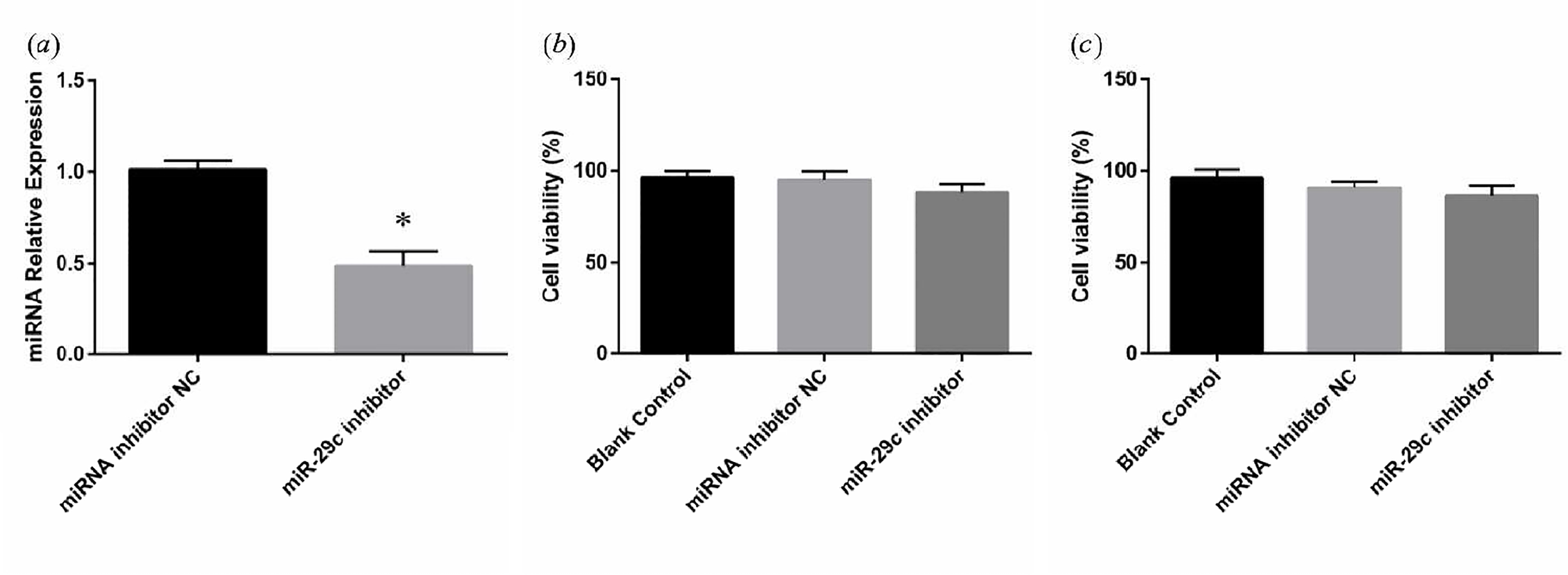

Supplement: Supplementary file 1 — Figure S1. Effects of miR-29c inhibitor transfection on GC-2 cell apoptosis. (a) The relative expression of miR-29c was measured by quantitative RT-PCR using U6 as the internal control. (b) MTT assay was performed to evaluate cell viability after miR-29c inhibition. Cells without transfection were considered blank controls. (c) CCK-8 assay was performed to evaluate cell viability after miR-29c inhibition. Cells without transfection were considered blank controls. All the results excluded the possibility of the interfering effect of miR-29c (Sa, 1 vs 0.5; Sb, 96.9 and 95.37% vs 90.73%; Sc, 96.9 and 90.73% vs 87.64%). All data represent the mean ± SEM of at least three independent experiments (*P < 0.05). (PNG 453 kb) [file 12958_2019_506_MOESM1_ESM.png]
