# Supplementary material for: Downregulation of miR-322 promotes apoptosis of GC-2 cell by targeting Ddx3x
Source: Reprod Biol Endocrinol. 2019 Aug 5;17:63. doi: 10.1186/s12958-019-0506-7 (PMC6683552; doi:10.1186/s12958-019-0506-7)
Supplement: Supplementary file 2 — Table S1. Sequences of primers for quantitative RT-PCR. (DOCX 43 kb) [file 12958_2019_506_MOESM2_ESM.docx]

**Table S1. Sequences of primers for quantitative RT-PCR**

| **miRNA/gene** |  | **Sequences** |
| --- | --- | --- |
| **mmu-miR-322** | **Reverse** | **5’-GTCGTATCCAGTGCAGGGTCCGAGGT**  **ATTCGCACTGGATACGACTCCAAA-3’** |
|  | **Forward Primer** | **5’-ATCCAGTGCAGGGTCCGAGG-3’** |
|  | **Reverse Primer** | **5’-CCGGCGCAGCAGCAATTCATGT-3’** |
| **mmu-miR-29c** | **Reverse** | **5’-GTCGTATCCAGTGCAGGGTCCGAGGT**  **ATTCGCACTGGATACGACTAACCG-3’** |
|  | **Forward Primer** | **5’-ACGCGCGCGTAGCACCATTTGAAAT-3’** |
|  | **Reverse Primer** | **5’-ATCCAGTGCAGGGTCCGAGG-3’** |
| **U6** | **Reverse** | **5’-AACGCTTCACGAATTTGCGT-3’** |
|  | **Forward Primer** | **5’-CTCGCTTCGGCAGCACA-3’** |
|  | **Reverse Primer** | **5’-TGGTGTCGTGGAGTCG-3’** |
| **Caspase-3** | **Forward Primer** | **5’-GTCTGACTGGAAAGCCGAAAC-3’** |
|  | **Reverse Primer** | **5’-GACTGGATGAACCACGACCC-3’** |
| **Caspase-9** | **Forward Primer** | **5’-GAGGTGAAGAACGACCTGACTG-3’** |
|  | **Reverse Primer** | **5’-CTCAATGGACACGGAGCATC-3’** |
| **Caspase-8** | **Forward Primer** | **5’-CTTGAAGGAAGGGAAGAGTTGC-3’** |
|  | **Reverse Primer** | **5’-CACTGTCTTGTTCTCTTGGCGA-3’** |
| **Bax** | **Forward Primer** | **5’-GCCTTTTTGCTACAGGGTTTCAT-3’** |
|  | **Reverse Primer** | **5’-TATTGCTGTCCAGTTCATCTCCA-3’** |
| **Bcl-2** | **Forward Primer** | **5’-TGACTTCTCTCGTCGCTACCGT-3’** |
|  | **Reverse Primer** | **5’-CCTGAAGAGTTCCTCCACCACC-3’** |
| **Ddx3x** | **Forward Primer** | **5’-TACAGCAAGCAAAGGGCGTT-3’** |
|  | **Reverse Primer** | **5’-ACCAGCGACTATTTCCACCTCT-3’** |
| **Uba1** | **Forward Primer** | **5’-CCAACCAACGGAATGGCGAAG-3’** |
|  | **Reverse Primer** | **5’-CCTGAGACAAGGACGCTGGAT-3’** |
| **Rad23b** | **Forward Primer** | **5’-TGACAAAACCCAAAGCAGTGAC-3’** |
|  | **Reverse Primer** | **5’-GAATCTCCTGGTGTACTGTCAGC-3’** |
| **β-actin** | **Forward Primer** | **5’-GTGACGTTGACATCCGTAAAGA-3’** |
|  | **Reverse Primer** | **5’-GTAACAGTCCGCCTAGAAGCAC-3’** |
